# Self-efficacy of PLHIV for self-management at the University of Gondar Comprehensive Specialized Hospital, Northwest Ethiopia: a cross-sectional study

**DOI:** 10.1186/s12875-024-02502-5

**Published:** 2024-07-15

**Authors:** Abdisa Gemedi Jara, Faisel Dula Sema, Masho Tigabe Tekele, Asrat Elias Ergena, Eyayaw Ashete Belachew, Amensisa Hailu Tesfaye, Abenezer Melaku Tafese, Banchamlak Teferi Mekonen, Saron Naji Gebremariam, Endalamaw Aschale Mihiretie, Eden Abetu Mehari

**Affiliations:** 1 Department of Clinical Pharmacy, School of Pharmacy, College of Medicine and Health Sciences, University of Gondar, P.O. Box 196, Gondar, Ethiopia; 2 Department Pharmaceutical Chemistry, School of Pharmacy, College of Medicine and Health Sciences, University of Gondar, P.O. Box 196, Gondar, Ethiopia; 3 Department of Environmental and Occupational Health and Safety, Institute of Public Health, College of Medicine and Health Sciences, University of Gondar, P.O. Box 196, Gondar, Ethiopia; 4 Department of Internal Medicine, School of Medicine, College of Medicine and Health Sciences, University of Gondar, P.O. Box 196, Gondar, Ethiopia; 5 Clinical Pharmacy Unit, Department of Pharmacy, College of Medicine and Health Sciences, Bahir Dar University, Bahir Dar, Ethiopia

**Keywords:** PLHIV, HIV/AIDS, Self-efficacy

## Abstract

**Background:**

Self-efficacy is the ability to execute, and it is a critical predictor of health-related outcomes among people living with human immunodeficiency virus (PLHIV). Self-efficacy directly determines treatment outcome. However, there is no evidence on the self-efficacy of PLHIV for self-management in Ethiopia. Currently, HIV is considered a manageable chronic disease. However, the burden remains high despite all the taken measures.

**Objectives:**

This study aimed to assess the self-efficacy of PLHIV for self-management at the University of Gondar Comprehensive Specialized Hospital (UOGCSH), northwest Ethiopia, 2022.

**Methods:**

A cross-sectional study was conducted on PLHIV selected by a systematic random sampling technique using an interviewer-administered questionnaire at the UOGCSH from August 10 to September 30, 2022. The data was entered and analyzed using the Statically Package for Social Science version 25.0. Categorical variables were summarized as frequency (percentage) of the total. Both descriptive and inferential statistics, such as the Kruskal-Wallis H test and Mann-Whitney U test were performed to detect difference. *P*-value < 0.05 was considered to indicate statistically significance.

**Results:**

Overall, 405 PLHIV participated in the study, giving a 96% response rate. The overall median (Interquartile range) self-efficacy score of PLHIV for self-management was 22 (4) and 67.4% of the PLHIV self-efficacy score was above the median. A statistically significant difference was detected between the social support groups (χ^2^ (2) = 37.17, *p* < 0.0001), education background (U = 10,347, Z = 2.279, *P* = 0.023, *r* = 0.113), living conditions (U = 12,338, Z = 2.457, *P* = 0.014, *r* = 0.122) and medication adherence (U = 9516.5, Z = 3.699, *P* < 0.0001, *r* = 0.184).

**Conclusion:**

Most participants’ self-efficacy score was above the median. Statistically significant differences in self-efficacy were observed based on individual, environmental, and clinical factors. We suggest training and workshops for healthcare workers and the hospital and adherence support groups should work to improve the self-efficacy of PLHIV.

## Background

Human Immunodeficiency Virus (HIV) is a disease that targets the human immunity system (CD4 cells) and reduces the body’s defense system. Since the start of the epidemic, 40.1 million people died from acquired immune deficiency syndrome (AIDS). In 2021, 38.4 million and 513,836 people lived with human immunodeficiency virus (PLHIV) in the world and Ethiopia, respectively [1, 2]. Currently, there is no definitive treatment for HIV. However, because of the measures taken regarding it is prevention, diagnosis, and treatment, currently, HIV is considered a manageable chronic disease.

The word self-efficacy was first defined by Bandura A. 45 years ago [3] as individuals’ perception regarding the adaptation of changes that have to be executed to achieve a better health outcome [4]. In addition, self-efficacy is a set of responses toward stated goals and it is a part of the self-regulation process through which individuals manage resources and behaviors to achieve the desired goal [5]. Self-efficacy results from a combination of individual, behavioral, and environmental factors [6]. Overall, individual self-efficacy determines health-related goals, effort to reach the goal, duration of activity to achieve the goal, and disease outcomes [5], and good self-efficacy is key in the treatment and self-management of HIV/AIDs [7].

Available evidence shows that self-efficacy promotes better disease management, symptom control, well-being, and health-related quality of life [8–11]. Furthermore, self-efficacy correlated with self-management among PLHIV [12, 13], which is the direct active participation of the patient in the treatment process and it includes disease management, lifestyle modification, and dealing with chronicity of the diseases [14]. In a similar fashion to other diseases, self-efficacy is associated with health promotion [3, 15], positive coping strategy [16], HIV treatment adherence [17], perceived social support [18], and it is a direct predictor of viral load among PLHIV [19]. In addition, better self-efficacy increases acceptance of healthcare professionals’ recommendations and counseling [18]. Due to the reasons mentioned above, self-efficacy is considered an important and critical predictor of health-related outcomes among PLHIV [17]. The poorer the self-efficacy, the worse the health-related outcomes tend to be [4, 14, 15].

The level of self-efficacy varies across places and disease conditions. A study conducted in South Korea among patients with chronic diseases revealed that the study participants had a moderate level of self-efficacy for self-management (mean ± SD, 6.36 ± 1.17) [20]. Another study conducted in Hubei Province among PLHIV showed low self-efficacy (mean ± SD, 22.7 ± 7.8) [21]. In Ethiopia, studies have showed that 52.5% and 46.2% of participants had good self-efficacy to ward self-management of diabetes mellitus [22] and preventive measures for COVID-19 [23], respectively. There is a lack of evidence about the self-efficacy of PLHIV for self-management in Ethiopia, which provides a real picture of the self-efficacy of PLHIV, a gap that has to be addressed and serve as a baseline. To the best of our knowledge, there are no studies conducted in Ethiopia that determine the self-efficacy of PLHIV for self-management, for this reason, it is essential to determine the self-efficacy of PLHIV for self-management. This study aimed to assess self-efficacy for self-management and associated factors among PLHIV at the University of Gondar Comprehensive Specialized Hospital (UOGCSH). This study will provide baseline data on PLHIV self-efficacy for self-management, it will initiate and help researchers to further assess the impact of self-efficacy and related health outcomes.

## Materials and methods

### Study design and setting

A cross-sectional study was conducted at the UOGCSH antiretroviral therapy (ART) clinic from August 10 to September 30, 2022. UOGCSH resides in Gondar city, Amhara Region, Ethiopia. This city is found 738 km from Addis Ababa (the capital city of Ethiopia). UOGCSH was established by the Federal Ministry of Health in 1954 and currently, it serves as a referral hospital for more than 8 million urban and rural inhabitants. The hospital has approximately 1000 beds for inpatients and fourteen outpatient departments. The ART clinic is an outpatient department and has a total of approximately 5500 registered retroviral infection (RVI) patients as of September 2022.

### Population of the study

All RVI patients who had regular follow-ups at the UOGCSH ART clinic were the source population. RVI patients who visited the ART clinic during the data collection period were considered as the study population.

### Eligibility criteria

Patients available during the data collection period, willing to participate, and age older than 18 years were included in the study. Patients who were not able to care for themselves, patients who visited the ART clinic for emergency conditions, or patients with difficulty of communication were excluded. In addition, since the study did not use insight measurements, patients with medically confirmed psychiatric diagnoses were also excluded from the study.

### Sample size determination and sampling technique

The sample size was determined using a single population proportion formula $\text{N}=\frac{{\left(\frac{\text{z}{\upalpha }}{2}\right)}^{2}P(1-p)}{{\left(d\right)}^{2}}$. where, **N**= sample size, P= proportion, and **d**= margin of error. The estimated prevalence of self-efficacy among PLHIV was 50% and the margin of error was 5% at a 95% confidence level $\text{N}=\frac{{\left(1.96\right)}^{2}0.5(1-0.5)}{{\left(0.05 \right)}^{2}}$= 384. After consideration of the 10% nonresponse rate, the total sample size was 422.

Participants were selected using a systematic random sampling technique. The ART clinic data showed that 1680 PLHIV had appointment during the data collection period. Three was a randomly selected number to select the first participant and four was the skipping interval. The sampling started from roll number 3, and then, every 4th participant was included in the study. PLHIV who discontinued the interview after they gave written informed consent and started the interview, were considered as non-respondent. $k=\frac{Nv}{Nf}=\frac{1680}{422}=3.98\sim4$

where, Nv = Number of PLHIV expected to visit the ART clinic during the data collection period and Nf = total sample size calculated for this study.

### Study variables

Self-efficacy was the dependent variable. However, gender, educational status, job status, living conditions, marital status, residence, comorbidity, self-reported drug side effects, route of infection, adherence to ART medications, social support, and other HIV/AIDS-related factors were treated as independent variables (Table 1).

**Table 1:** Socio-demographic, clinical factors and environmental characteristics, University of Gondar Comprehensive Specialized Hospital, 2022

Variables		Frequency	Percentile (%)
Age	Above mean	184	45.4
Mean ± SD	41.30 ± 12.36		
Sex	Male	131	32.3
	Female	274	67.7
Education level	Illiterate	75	18.5
	Literate	330	81.5
Job	Employed	131	32.3
	Self employed	122	30.1
	jobless	152	37.5
Living condition	Live alone	95	23.5
	Live with family	310	76.5
Marital status	Single	62	15.3
	Married	155	38.3
	Divorced	131	32.3
	Widowed	57	14.1
Residency	Rural	38	9.4
	Urban	367	90.6
Comorbidity	Yes	65	16
	No	340	84
Do you have drug side effect	Yes	44	10.9
	No	361	89.1
Transmission route	Sexual intercourse	220	54.3
	MTCT*	25	6.2
	Accidentally by sharp material	40	9.9
	I don’t remember	120	29.6
I try to have a plan for SM of emotional distress	Yes	237	58.5
	No	168	41.5
I am familiar with how to manage HIV related symptoms	Yes	161	39.8
	No	244	60.2
Have you set a goal in the process of your HIV therapy	Yes	255	63
	No	150	37
Social support	Poor support	106	26.2
	Intermediate support	149	36.8
	Strong support	150	37
Adherence	Adherent	326	80.5
	Non-adherent	79	19.5
Did you supported by an adherence support group	Yes	83	20.5
	No	322	79.5
Do you think the counseling you got was adequate for the next HIV treatment	Yes	311	76.8
	No	94	23.2
Have you been encouraged to disclose your HIV status	Yes	267	65.9
	No	138	34.1
I have accepted that HIV is a chronic condition that can be managed	Yes	358	88.4
	No	47	11.6
My HIV doctor and I have a good relationship	Yes	261	64.4
	No	144	35.6

MTCT: Mother to child transmission*, HIV: human immunodeficiency virus

### Data collection instrument, procedure, and quality control

The interviewer-administered questionnaire was prepared in English by reviewing the available literature and validated tools [12, 20, 24–26]. Furthermore, the questionnaire was translated into Amharic (a local language) for a better understanding of the study participants and back-translated to English to minimize potential translation errors.

The questionnaire contains 8 items and is scored on a 3-point Likert scale (1 = disagree, 2 = neutral, and 3 = agree) to measure the self-efficacy of PLHIV for self-management. The reverse scoring technique was used for question numbers S1, S2, S6 and S7 (Table 2). The total score ranges from 8 to 24, with higher scores reflecting greater self-efficacy [25]. Furthermore, social support was measured by using the Oslo3 social support scale (OSS-3). On the OSS-3, the statement “how many people are so close to you that you can count on them if you have a series problem”, accounts for 4 points if the patient responds above 5, 3 points for 3–5, 2 points for 1 or 2 and 1 point for none. The other 2 questions have the lowest score of 1 and a maximum score of 5. The total score ranges from 3 to 14, and patients who score 12–14, 9–11, or 3–8 points were classified as having strong social support, intermediate social support, or poor social support, respectively [24].

**Table 2:** Self-efficacy of PLHIV for self-management at University of Gondar Comprehensive Specialized Hospital, 2022

	Self-efficacy measurement tools	Disagree *N* (%)	Neutral *N* (%)	Agree *N* (%)
S1	It is difficult for me to find effective solution *	298(73.6)	43 (10.3)	64 (15.8)
S2	I find my effort ineffective to change *	202 (49.9)	114(28.1)	89 (22)
S3	I handle well my self-regarding my HIV infection	25 (6.2)	13 (3.2)	367 (90.6)
S4	I succeed in the project to manage my HIV infection	18 (4.4)	21 (5.2)	366 (90.4)
S5	I am able to manage my HIV as well as other people	22 (5.4)	14 (3.5)	369 (91.1)
S6	Typically my plan to HIV don’t work out well*	340 (84)	28 (6.9)	37 (9.1)
S7	No matter how hard I try my HIV do not turn the way I would like *	305 (75.3)	48 (11.9)	52 (12.8)
S8	I am generally able to accomplish my HIV infection goal	33 (8.1)	30 (7.4)	342 (84.4)

N: frequency, HIV: Human immunodeficiency virus, Reverse scoring was used (*)

The data were collected by two pharmacists (B. pharm) after training was given on the objective of the study, methodology, data collection method, confidentiality of information, participants’ rights, and ethical aspects. To ensure the clarity, wording, logical sequence, and reliability of the tool, the questionnaire was pretested on 50 RVI patients at UOGCSH before the actual data collection began and the data was not considered for the final analysis. The reliability statistical test (Cronbach’s alpha) for self-efficacy and social support tools used in this study was 0.877 and 0.667, respectively. After appropriate training was given to the supervisor, the collected data was reviewed and checked regularly for completeness, accuracy, and consistency by the supervisor and principal investigators.

### Data processing and analysis

The data that passed quality control were entered and analyzed by using the Statically Package for Social Science (SPSS) version 25.0. Descriptive statistics, Kruskal-Wallis H test, and Mann-Whitney U test were performed. Multicollinearity was assessed using the variance inflation factor; the maximum variance inflation factor obtained was < 10. A Kolmogorov-Smirnova statistical test was used to test the normality of the data (normally distributed when the *P*-value > 0.05). Normally distributed and skewed continuous variables were expressed as the mean (standard deviation) and median (Interquartile range), respectively. Categorical variables were summarized as frequency (percentage) of the total. As the data was skewed, the self-efficacy score was reported by median (IQR). Mann-Whitney U test was performed to detect the difference in self-efficacy for variables with one degree of freedom. In addition, the effect size was determined for variables with significant median self-efficacy differences on the Mann-Whitney U test. Whereas, Kruskal-Wallis H test was performed to detect the difference in self-efficacy for variables with two or more degrees of freedom. For variables with significant median self-efficacy differences according to the Kruskal-Wallis H test, pairwise multiple comparisons were performed. *P*-value < 0.05 was used to indicate statistical significance.

## Results

### Sociodemographic status of participants

In this study, 405 PLHIV participated, giving a 96% of response rate. The majority of the participants were females (67.7%) and literate (81.5%). Approximately one-third (37.5%) of the participants were jobless. Furthermore, only one-tenth (10.9%) of the participants have self-reported drug side effects. Of all the participants, less than half were not familiar with the management of HIV/AIDS-related symptoms (39.8%) and did not set goals for their HIV/AIDS treatment (37%). Around a quarter (26.2%) of the participants had poor social support. More than three-fourths of the participants were adherent to their ART medications (80.5%) and think the counseling they get during ART clinic visits is adequate (76.8%). During their routine ART clinic follow-ups, around two-thirds of the study participants were encouraged to disclose their HIV status (65.9%) and had a good relationship with their HIV/AIDS doctors (64.4%) (Table 1).

### Self- efficacy of PLHIV for self-management

**Fig. 1:**
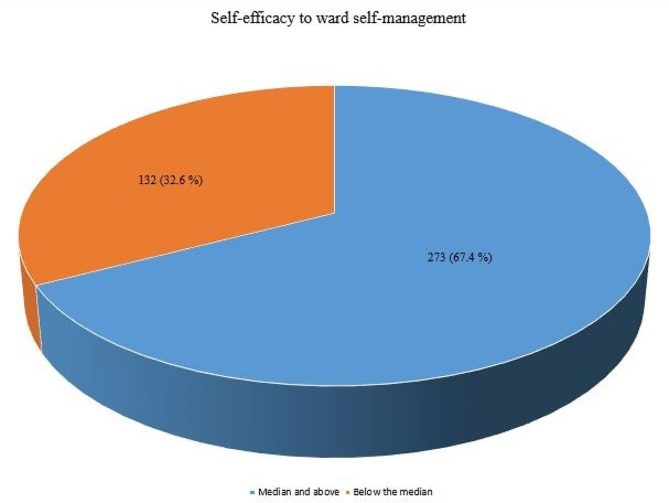
Self-efficacy of PLHIV for self-management at University of Gondar Comprehensive Specialized Hospital, 2022

This study revealed that participants had better self-efficacy on specific measurement questions. Among all participants, more than three-quarters handled themselves well regarding HIV infection (90.6%), succeeded in the project of managing HIV infection (90.4%), and were able to manage HIV as well as other people (91.1%) (Table 2). The overall median (IQR) self-efficacy score was 22 (4) and 67.4% of the study participants’ self-efficacy score was above the median (Fig. 1).

A Kruskal-Wallis H test showed that there was a statistically significant difference in self-efficacy between the different social support groups (χ^2^ (2) = 37.17, *p* < 0.0001) and according to pairwise comparison, a significant difference was observed in the self-efficacy score among PLHIV who had poor social support when they compared with participants with intermediate and strong social support. However, there was no group difference between PLHIV with intermediate and strong social support (Table 3).

**Table 3:** Kruskal-Wallis-H test for self-efficacy at the University of Gondar Comprehensive Specialized Hospital, 2022

Variable		Mean rank Square	Test statistics (X^2^), (df)	*P*-value
Job	Employed	177.87	4.893, (2)	0.087
	Self employed	210.31		
	jobless	213.10		
Marital status	Single	170.19	7.182, (3)	0.066
	Married	215.26		
	Divorced	206.37		
	Widowed	197.61		
Transmission route	Sexual intercourse	205.60	6.372, (3)	0.095
	MTCT	159.06		
	Accidentally by sharp material	230.48		
	I don’t remember	198.22		
Social support	Poor support	145.43	37.17, (2)	**< 0.0001**
	Intermediate support	218.67		
	Strong support	228.11		

MTCT: Mother to child transmission, (X^2^): Chi-square test, (df): Degree of freedom

The Mann-Whitney U test which was used to detect difference in self-efficacy scores according to the available groups revealed significant difference in self-efficacy among the literates and illiterate individuals (U = 10,347, Z = 2.279, *P* = 0.023, *r* = 0.113), those who lived alone and those who lived with family (U = 12,338, Z = 2.457, *P* = 0.014, *r* = 0.122), those who were adherent to ART medication and nonadherent individuals (U = 9516.5, Z = 3.699, *P* < 0.0001, *r* = 0.184) and those who set goals during the treatment process and who did not set goals (U = 13,702, Z = 4.898, *P* < 0.0001, *r* = 0.24) (Table 4).

**Table 4:** Mann-Whitney U test of self-efficacy at University of Gondar Comprehensive Specialized Hospital

Variable		Mean rank square	Mann-Whitney U test	Z-score	*P*-value	Effect size (*r*)
Age	Above mean	208.10	19394.5	-0.821	0.412	
Mean ± SD	41.30 ± 12.36					
Sex	Male	196.94	17152.5	− 0.741	0.459	
	Female	205.9				
Education level	Illiterate	175.93	10,347	-2.279	**0.023**	**0.113**
	Literate	209.15				
Living condition	Live alone	177.87	12,338	-2.457	**0.014**	**0.122**
	Live with family	210.70				
Residency	Rural	182.81	4866.5	-0.99	0.322	
	Urban	204.56				
Comorbidity	Yes	179.36	9513.5	-1.826	0.068	
	No	207.52				
Do you have drug side effect	Yes	170.80	6525	-1.986	**0.047**	**0.1**
	No	206.93				
I try to have a plan for SM of emotional distress	yes	211.54	17,883	-1.793	0.073	
	No	190.95				
I am familiar with how to manage HIV related symptoms	Yes	225.48	16023.5	-3.225	**0.001**	**0.16**
	No	188.17				
Have you set a goal in the process of your HIV therapy	Yes	224.27	13,702	-4.898	**< 0.001**	**0.24**
	No	166.85				
Adherence	Adherent	213.31	9516.5	-3.699	**< 0.001**	**0.18**
	Non-adherent	160.46				
Did you supported by an adherence support group	Yes	199.95	13,110	-0.273	0.785	
	No	203.79				
Do you think the counseling you got was adequate for the next HIV treatment	Yes	209.60	12564.5	-2.121	**0.034**	**0.105**
	No	181.16				
Encourage to disclose your HIV status	Yes	215.14	15,182	-2.983	**0.003**	**0.15**
	No	179.51				
I have accepted that HIV is a chronic condition that can be managed	Yes	210.35	5780.5	-3.585	**< 0.001**	**0.18**
	No	146.99				
My HIV doctor and I have a good relationship	Yes	226.59	13581.5	-4.748	**< 0.001**	**0.24**
	No	178.34				

SD: standard deviation, HIV: human immunodeficiency virus

## Discussion

This study is the first to determine the self-efficacy of PLHIV for self-management and its determinants in Ethiopia. The median (IQR) self-efficacy score of PLHIV for self-management was 22 (4). A total of 67.4% of the study participants’ self-efficacy for self-management was above the median, and this result was greater than that of study conducted on self-efficacy for COVID-19 prevention in Ethiopia [23]. This difference could be because PLHIV have more years lived with HIV/AIDS than with COVID-19 and available evidence indicates that self-efficacy increases with the number of years lived with the medical condition [20, 27]. Even though, self-efficacy is higher, it is better to implement self-efficacy programs, heath education regarding self-efficacy and behavioral changes that increase the self-efficacy of PLHIV. Which in turn leads to positive treatment outcome.

In addition, this study compared self-efficacy across sociodemographic characteristics, adherence, social support, and clinical factors among PLHIV. A statistically significant difference in self-efficacy was observed among literate and illiterate PLHIV. This difference might be observed due to difference in cognitive appraisal ability between illiterate and literates. This finding is in line with a study conducted in Korea [20]. Another study conducted among diabetes patients in Nigeria also reported that educational status was significantly associated with self-efficacy [28]. This is because self-efficacy is determined by literacy level, and improving literacy can increase the self-efficacy for self-management [29, 30]. In addition, being illiterate leads to poor self-management, which is directly associated with self-efficacy [31].

This study revealed that there was a statistically significant difference in the self-efficacy of PLHIV for self-management between participants who lived with their family and those who lived alone. This might be observed due to support and motivation difference in day-to-day life. A similar result was reported in studies conducted among diabetes mellitus patients in Ethiopia [22] and diabetes patients in other countries [32, 33]. A randomized controlled trial of family-oriented self-management to improve self-efficacy also reported that self-efficacy is better among patients who live with family than among those who live alone [34]. Since patients who live with their families received additional support, self-efficacy can be determined by the living conditions of the patients [35].

A statistically significant difference in self-efficacy was observed among PLHIV who had and those who did not had self-reported drug side effects. Another statistically significant difference in self-efficacy was detected among PLHIV who were familiar with and not familiar with the management of HIV/AIDS-related symptoms. Furthermore, a statistically significant difference in self-efficacy was observed based on ART medication adherence. This might be because side effects, managing HIV/AIDS-related symptoms and ART medication adherence determine the health status of PLHIV [3, 36]. It is important to increase the knowledge of PLHIV about side effects, the management of HIV/AIDS-related symptoms, and adherence to ART medication.

This study revealed a statistically significant difference in self-efficacy between PLHIV who set goals in the treatment process and those who did not. This difference might be observed due to setting goals guides them to ward specific activities. Another review of university students’ self-efficacy reported that goal setting increases self-efficacy and performance [37]. J.E. Maddux also reported that goal seating or visualization determine self-efficacy [38]. A statistically significant difference was observed in self-efficacy between PLHIV who have adequate counseling on HIV/AIDS treatment and those who did not. In addition, A statistically significant difference was observed in self-efficacy among PLHIV encouraged on disclosing HIV/AIDS status and those who did not. This might be due to having adequate counseling on HIV/AIDS treatment and encouragement of HIV/AIDS status disclosure increases vicarious experience developed by PLHIV including self-efficacy. In addition, this can lead to the creation of role models, having positive role models that encourage and guide increases the self-efficacy of PLHIV to ward self-management [3].

A statistically significant difference in self-efficacy was also observed between PLHIV who had a good relationship with ART clinic health care professionals and PLHIV who did not. Similarly, a study conducted among patients with chronic diseases in South Korea reported that self-efficacy is determined by patients’ relationships with healthcare professionals [20]. In addition, studies conducted in Palestine and the United States of America have shown that patients with good patient physician communication have better self-efficacy [39, 40]. Having good relationships with health care professionals increases counseling acceptance by PLHIV and this increases knowledge of the disease condition, which leads to an increase in confidence and self-efficacy for self-management of PLHIV.

Even though, the environmental factors affect the self-efficacy it is not the solely predictor. Vicarious and mastery experience, emotional status and difference in cognitive processing might have impact on the self-efficacy [3].

The finding of this Study helps ART clinic health care professional to intervene based on identified gaps and helps social support groups to include self-efficacy skills in their education programs. In collaboration with other findings this study will help policymakers and the Minister of health to incorporate self-efficacy programs in the management of HIV/AIDS in Ethiopia. In addition, since the self-efficacy of PLHIV in Ethiopia was unknown, this finding will serve as a baseline and initiate researchers to further investigate self-efficacy of PLHIV.

### Strengths and limitations of the study

This study is the first in its type and was conducted with an adequate sample size. Despite the authors’ effort, this study is not without limitations. The single-center nature of the finding may limit the generalizability of the finding, and due to the intrinsic nature of a cross-sectional study, the cause-and-effect relationships between the independent and dependent variables cannot be determined. Since self-report measures used to asses self-efficacy and adherence of PLHIV, the response of the participants might be subjected to recall bias and social desirability bias. The fact that Psychiatric disorders are common among PLHIV excluding psychiatric patients may also limits the generalizability of these finding.

## Conclusion

The majority of the PLHIV self-efficacy scores for self-management were above the median. Our results showed that literacy level, living conditions, drug side effects, HIV/AIDS-related symptom management, social support, medication adherence, goal setting, counseling, acceptance of HIV/AIDS as a chronic disease, and strength of relationships with ART clinic health care professionals are associated with self-efficacy. It is better to prepare training and workshops for ART clinic health care workers and social support groups to improve the self-efficacy of PLHIV. In addition, hospital, adherence support groups, and PLHIV should work on modifiable factors to improve self-efficacy for self-management.

## Data Availability

The datasets used and/or analyzed during the current study are available from the corresponding author upon reasonable request.

## Abbreviations

ART: Antiretroviral Therapy
RVI: Retroviral infection
HIV/AIDS: Human immunodeficiency virus/ acquired immune deficiency syndrome
IQR: Interquartile range
PLHIV: People Living with Human Immunodeficiency Virus
UOGCSH: University of Gondar Comprehensive Specialized Hospital
USA: United States of America
